# Approaches to Macroevolution: 1. General Concepts and Origin of Variation

**DOI:** 10.1007/s11692-017-9420-0

**Published:** 2017-06-03

**Authors:** David Jablonski

**Affiliations:** Department of Geophysical Sciences, University of Chicago, 5734 South Ellis Avenue, Chicago, IL 60637 USA

**Keywords:** Evolutionary developmental biology, Contingency, Hierarchy, Diversification, Disparity, Evolutionary novelty, Paleobiology

## Abstract

Approaches to macroevolution require integration of its two fundamental components, i.e. the origin and the sorting of variation, in a hierarchical framework. Macroevolution occurs in multiple currencies that are only loosely correlated, notably taxonomic diversity, morphological disparity, and functional variety. The origin of variation within this conceptual framework is increasingly understood in developmental terms, with the semi-hierarchical structure of gene regulatory networks (GRNs, used here in a broad sense incorporating not just the genetic circuitry per se but the factors controlling the timing and location of gene expression and repression), the non-linear relation between magnitude of genetic change and the phenotypic results, the evolutionary potential of co-opting existing GRNs, and developmental responsiveness to nongenetic signals (i.e. epigenetics and plasticity), all requiring modification of standard microevolutionary models, and rendering difficult any simple definition of evolutionary novelty. The developmental factors underlying macroevolution create anisotropic probabilities—i.e., an uneven density distribution—of evolutionary change around any given phenotypic starting point, and the potential for coordinated changes among traits that can accommodate change via epigenetic mechanisms. From this standpoint, “punctuated equilibrium” and “phyletic gradualism” simply represent two cells in a matrix of evolutionary models of phenotypic change, and the origin of trends and evolutionary novelty are not simply functions of ecological opportunity. Over long timescales, contingency becomes especially important, and can be viewed in terms of macroevolutionary lags (the temporal separation between the origin of a trait or clade and subsequent diversification); such lags can arise by several mechanisms: as geological or phylogenetic artifacts, or when diversifications require synergistic interactions among traits, or between traits and external events. The temporal and spatial patterns of the origins of evolutionary novelties are a challenge to macroevolutionary theory; individual events can be described retrospectively, but a general model relating development, genetics, and ecology is needed. An accompanying paper (Jablonski in Evol Biol 2017) reviews diversity dynamics and the sorting of variation, with some general conclusions.

## Introduction

Macroevolution, defined broadly as evolution above the species level, is thriving as a field. History, scale, and hierarchy are now entrenched in the evolutionist’s conceptual and analytical toolkit to an unprecedented degree, and paleontology and developmental biology are now more fully incorporated into evolutionary theory and analysis than at any time in the past century. Approaches to macroevolution involve the same fundamental components as evolutionary theory as a whole: the generation and sorting of variation (Jablonski 2000). Similarly, the process of evolution by natural selection, with its variation, interaction, and heritability triad, has a fundamental logic that applies across levels and scales. This paper and its companion (Jablonski 2017) reviews the two major engines of evolution—variation and sorting—at large spatial and temporal scales, and the hierarchical organization and dynamics of genealogical units. Under this approach to macroevolution, the origin and fates of major evolutionary novelties, the long-term evolutionary role of rare events ranging from the internal redeployment of gene regulatory networks to externally driven mass extinctions, and the potential for emergent properties or dynamics at different hierarchical levels, are key issues.

A multilevel, multi-scale view of evolution can be found in many classic sources, including Darwin’s *Origin* (see Gould 1977, 2002; Amundson 2005; Futuyma 2015). Such thinking receded with the burgeoning of population and quantitative genetics, but the 1970s and 1980s saw a renaissance in macroevolutionary theory and analysis that has been well-documented on the paleontological side by Sepkoski (2012; Sepkoski and Ruse 2009) and discussed in terms of evolutionary developmental biology by Amundsen (2005; see also Moczek et al. 2015; and Futuyma 2015 for a wide-ranging commentary). Whether these changes represent an overturning, an expansion, or a minor polishing of the neodarwinian theory of 50 years ago depends entirely on whose version of neodarwinism is used (compare, for example, Gould 2002; Jablonski 2007; Pigliucci and Müller 2010; Futuyma 2015; Laland et al. 2015).

Despite much progress, two major integrative efforts are still incomplete. One is the integration of paleontology and neontology. We lack a formal methodology for the developmental interpretation of the wealth of extinct phenotypes and their ecological, geographic, and temporal context, or the merging of phylogenetic data incorporating fossil and living taxa, and the attendant problems of merging phenotypic and molecular data, when so many clades are predominantly represented by one type or the other. A second work-in-progress is the integration of the two major strands noted above: the origin of variation on one hand, and its differential survival and reproduction on the other. Macroevolutionary researchers tend to focus on just one of these areas, but clearly both are as important at higher levels and large scales as they have long been recognized to be for microevolution. The split between origins and dynamics used to fall mainly along paleo/neo lines, but with the advent of comparative phylogenetic analysis the division tends to fall between evolutionary developmental biology on one hand, and historical biology in its many guises on the other.

Here, I will attempt to summarize these components and how they might fit together. I start with some basic concepts such as scale, hierarchy, and contingency, and then discuss macroevolutionary approaches to the origin of variation within and among clades. In a second paper (Jablonski 2017), I discuss macroevolutionary approaches to the sorting of variation, followed by some general questions and conclusions on a few of the most promising research directions.

## Macroevolutionary Concepts

### Scale and Hierarchy

The distinction between scale and hierarchy is fairly clear for biological systems (Jablonski 2007). Scale involves more or less arbitrary quantities of a given measure. We create nested units for time, space, weight, and so on, but these too are essentially arbitrary (how many yards in a mile?), and the units are categories, or classes in the philosophical sense: a gram of gold weighs as much as a gram of hydrogen. From a macroevolutionary perspective, scale may be most interesting in terms of whether evolutionary phenomena viewed on long temporal scales flow smoothly and predictably from those observed over the short term, and phenomena observed at the provincial, continental, or global scale similarly flow from those observed locally. In at least some instances they evidently do not. Empirical examples, important because they were not expected from dominant theories and models of the time, range from the morphological stasis or nondirectional random walks common in the fossil record at the 1–10 million-year timescale, rather than the sustained evolutionary transformations formerly expected in light of the evolutionary responsiveness of local populations on annual or decadal timescales (Hunt 2007a), to evidence that mass extinction events can qualitatively change survivorship patterns and thus re-direct evolutionary trajectories in ways not predicted from dynamics in calmer intervals (Jablonski 2005a). Such predictive failures, which need not occur in all times, places and clades to be relevant, do not *necessarily* require novel processes to operate at those scales, but at the very least indicate that macroevolutionary theory cannot consist of simple extrapolation of short-term, local models and empirical outcomes.

In contrast to scalar measures, many biological hierarchies involve nested entities—individuals in the philosophical sense—with distinctive properties at each level, such that events at each level can propagate upward to larger, more inclusive entities and downward to their constitutive components. However, one attribute of a nested hierarchy is asymmetry of effects: dynamics at lower levels need not be manifest at higher levels, whereas dynamics at higher levels always propagate downward (e.g., Salthe 1985; Valentine and May 1996). Thus, a parasitic DNA sequence might never proliferate to the point of reducing the fitness of the host organism, and many selectively driven changes in organismal phenotype may have little effect on the extinction probability of their entire species or clade relative to a sister group. But the probabilistic loss of certain species owing to their narrow geographic ranges will preferentially remove, by downward causation, the parasitic DNA sequences in their cells, and quite possibility an associated array of clade-specific organismic traits; decrease the probability of extinction risk for other species via enlarged geographic ranges, and many organismic traits unrelated to biogeography will be at lower extinction risk. As discussed further in Part 2, the cross-level decoupling of dynamics can be profound. Thus, deterministic selective forces driving changes in individual species can sum to effective randomness at the clade level (Raup 1977; Eble 1999; McShea and Brandon 2010; see also Huang et al. 2015 and Hopkins 2016 for empirical examples).

Several biological hierarchies have been defined, each with its own rules and implications, but the chief *conceptual* focus of macroevolutionary theory, and this review, is a genealogical hierarchy comprising genes, organisms, demes (genetically defined conspecific populations), species, and clades (Valentine 1973; Salthe 1985; Eldredge 1985, 1996; Valentine and May 1996; Gould 2002; Jablonski 2007; McShea and Brandon 2010; Scheiner 2010; Myers and Saupe 2013; Tëmkin and Eldredge 2015). Macroevolution is often *analyzed* using another hierarchy, that of formal taxonomy, frequently focused at the genus level, in part to reduce species-level sampling biases, but also as a rough proxy for ecological and functional diversity. Although taxonomic ranks are notoriously subjective, evidence is accumulating that genera, while imperfect, correspond sufficiently to genealogical units that they can often be used as proxies for low-level clades, i.e. monophyletic clusters of similar species (e.g. Jablonski and Finarelli 2009; Soul and Friedman 2015), and an analysis of genetic distances finds that the lower taxonomic ranks are more comparable across orders, classes, and phyla than generally assumed (Holman 2007).

Equally important for the use of higher taxa in macroevolutionary analyses, progress in modeling dynamics in the taxonomic hierarchy has derived insights from the frequency distribution of lower taxa within higher ones, including support for the claim that such distributions reflect natural evolutionary processes and not simply random agglomerations of low-ranked taxa (Holman 1985; Foote 2012; Maruvka et al. 2013; Humphreys and Barraclough 2014; Barraclough and Humphreys 2015). Sets of taxa ranked as orders, classes, and phyla exhibit different temporal and spatial patterns from those shown by species, genera, and families (see below and Jablonski 2017), a contrast seemingly consistent with suggestions that higher taxa reflect the origin of body plans while lower taxa reflect the dynamics of species formation and sorting, and that such differences may reflect different types of developmental changes (Valentine 1973, 2004; Davidson and Erwin 2009; Wagner 2014). More work in this area is needed, most notably on taxonomic ranking protocols that maximize the utility of taxa at different ranks for macroevolutionary analysis. Fossil taxa, almost all of which will forever lack sequence data, provide an essential window into the timing, location, and dynamics of past phenotypes, so that continued development of methods of integrating ancient phenotypes and genealogical units into larger ecological and phylogenetic frameworks are needed (see Hunt and Slater 2016 for a fine overview of this area).

### Macroevolutionary Currencies

Biodiversity can be measured in many ways; three macroevolutionary currencies that have received special attention are taxonomic richness, morphological disparity, and functional variety. These variables tend to be broadly correlated, and the use of higher taxa as rough proxies for disparity and functional variety has been validated repeatedly (Erwin 2007; Jablonski 2007; Berke et al. 2014; Chao et al. 2014), although such relationships tend to break down at finer timescales and among geographic regions (Jablonski 2008a; Brosse et al. 2013; Valiente-Banuet et al. 2014). Further, higher taxa tend to correspond to functional groups or adaptive zones for animals (e.g. Valentine 1973; Bambach 1985; Humphreys and Barraclough 2014), but major plant clades often split along reproductive lines with multiple convergences in phenotype and function (Donoghue 2008).

Each macroevolutionary currency has its own literature and methods, which impedes synthesis and the development of integrative models. Progress towards integration has begun with the recognition that different metrics and different data types (e.g. continuous and discrete characters in morphology) have different properties and thus relate to the others in complex but meaningful ways (Ciampaglio et al. 2001; Mason et al. 2013; Chao et al. 2014; Hetherington et al. 2015). For example, a clade that occupies a constant volume in morphospace (i.e. in a multidimensional space constructed from morphological variables with organisms plotted as points within that space) but diversifies taxonomically will decline in one key disparity measure, the mean pairwise distance among taxa, as taxa accumulate in the space (Foote 1996). Because the times and places where the different currencies are least correlated or most strongly nonlinear in their relationships—as in some major diversifications and extinctions, as discussed below—are of much interest, evolutionary models must go beyond the proxy assumption and treat the different currencies independently. The relationships among those currencies, and how they directly or indirectly affect one another, is thus a growth area in macroevolution.

### Contingency

The hierarchical framework is essential, but mechanistic models are difficult because macroevolutionary outcomes also depend heavily on contingency, in its multiple senses. The two main evolutionary applications of the term involve (a) chance, or unpredictable events, and (b), history, in terms of both intrinsic and extrinsic factors, i.e. the raw material provided by the biological entities under study at any hierarchical level, and their past environmental context (see Beatty’s seminal 2006 work on the multiple meanings of contingency; and Turner 2011, 2015; Erwin 2015a, 2016; Desjardins 2016). From a macroevolutionary standpoint, these concepts are complementary: “chance” implies that the same initial state can produce different outcomes, even if subjected to similar pressures, whereas “history” implies that different initial states can produce different outcomes, even under similar pressures. Paraphrasing Beatty (2006), prior states are necessary but insufficient to predict the outcome. However, even the “chance” events that cause evolutionary trajectories to diverge under similar conditions ultimately have a mechanistic driver; as already noted, in a hierarchical system deterministic processes at one level can produce effective randomness at another: the molecular changes behind an array of mutations for example, or the mating failures and genetic recombination in small populations that yield drift. Larger-scale unpredictable events such as the chance loss of species from a clade owing to severe weather events or even bolide impacts may be the macroevolutionary analogs. In that sense, chance and history are intimately related—today’s chance event is tomorrow’s initial state. (Erwin 2016 also comments briefly on contingency in a hierarchical framework.)

The challenge is to develop theory and models that illuminate the relative contribution of chance and historical events to macroevolutionary patterns, as they intertwine with and influence the other factors discussed below. Several approaches have been applied. The most straightforward procedure would simply be to run replicated, controlled experiments from the same starting point—impossible for metazoans over geologic timescales, but feasible for long-term laboratory populations (see for example the contingency effects inferred by Blount et al. 2008, 2012; Meyer et al. 2012; Blount 2016; and for a more expansive view, Bell 2016).

#### Macroevolutionary Lags and Contingency

Scaling up to the metazoan world, one indirect indicator of contingency is the perpetual difficulty in pinpointing key innovations, i.e. characters or character states that trigger taxonomic diversification. Although some traits appear to be closely, and even repeatedly, associated with diversifications, many cherished novelties, from pharyngeal jaws in teleosts to multicellularity in eukaryotes, are associated with prolific diversification in some clades but not others. Indeed, some striking traits seem never to promote diversification, as in the low diversity of flamingos and anteaters, two impressive divergences from ancestral phenotype. Still other diversifications are not associated with any recognizable evolutionary novelty. Further, when an association is apparent, some traits have proven to originate long before the diversifications once causally attributed to them. The evolution of, for example, elaborately cross-regulated gene networks, internal chemosymbionts, or endothermic metabolism should pay immediate dividends by promoting ecological dominance or prolific diversification, but although each feature is arguably integral to the success of one or more major groups, the fossil record indicates prolonged delays.

The temporal gap between the origin of a clade and its diversification or its rise to ecological dominance (two very different issues) has been termed a macroevolutionary lag (Jablonski and Bottjer 1990). Such lags are neglected tools for probing the relation between character evolution and clade dynamics, and provide a vehicle for the dissection of evolutionary contingency and both its causes and consequences. At least three broadly defined mechanisms have been proposed:

1. *Artifacts*, which fall into two categories. One is simply exponential diversification, with its inherent low-diversity lag phase—a proposition testable through modeling (Patzkowsky 1995). The other is sampling, both on the paleontological side owing to collection or preservational gaps, and on the phylogenetic side with the inability to access extinct stem taxa and phenotypes. Increasingly robust methods have developed for quantifying paleontological sampling failure (e.g. Foote 2010), although spatial variation remains a difficult issue (e.g. Valentine et al. 2013; Johnson et al. 2015). In phylogenies of extant organisms, the ability to infer close associations between a putative key innovation and diversification (and for that matter, the ability of infer ancestral character states in general) is inversely related to the length of internal branches linking phenotypically homogenous clades of extant organisms (Ané 2008). This escalating uncertainty derives from the number and disparity of extinct taxa bearing unknown character states within the unsampled portion of the tree. Improved integration of fossil and molecular data will sharpen inferences on the timing and phenotypic foundation of diversifications.
2. *Intrinsic changes* Many “key innovations” are actually assembled in a chain of derived characters, so that the most striking phenotypic changes may have been necessary but not sufficient to promote diversification. This stepwise model implies that highly diverse clades will often be associated with low-diversity sisters that share many of their attributes –- eukaryotes, most of the major branches of plant evolution, mammals, and the later diversifications of irregular sea urchins are likely examples (Butterfield 2015; Donoghue 2005; Luo 2007; Hopkins and Smith 2015; Donoghue and Sanderson 2015 call these novelty chains “synnovations” and provide additional examples). Traits that alter speciation rates or extinction rates, for example via geographic range sizes or genetic population structures, can also trigger diversifications long after more dramatic organismic traits are in place.
3. *Extrinsic events* Entry into a region that is vacant or occupied by inferior competitors or ineffective predators can provide belated “key opportunities” (Moore and Donoghue 2007, 2009; Schenk et al. 2013; see also Stroud and Losos 2016). On geologic timescales such opportunities can also occur in situ with changing conditions, from elimination of competitors in mass extinctions (Jablonski 2007) to expansion of potential habitat owing to climate change (e.g. Near et al. 2012 and Hu et al. 2016 on notothenioid fish). Even the Cambrian Explosion of metazoans has been viewed in terms of a macroevolutionary lag, perhaps linked to the rise in atmospheric oxygen (Erwin et al. 2011; Tweedt and Erwin 2015; see also Marshall and Valentine 2010; Erwin and Valentine 2013; and Knoll 2011 on the lag between the origin of proto-embryophytes and the Devonian Explosion of crown-group seed plants and ferns). The frequency of macroevolutionary lags and other poor correspondences between character evolution and taxonomic diversification suggest that changes in *both* extrinsic conditions and intrinsic traits are often (almost always?) required for prolific diversifications or radiations, and can be effective even when the intrinsic and extrinsic events are separated temporally (Bouchenak-Khelladi et al. 2015; Donoghue and Sanderson 2015 call these synergistic combinations of intrinsic and extrinsic factors “confluences” and again provide further examples).

#### Convergence and Contingency

Macroevolutionary lags seem to epitomize the role of contingency in evolution. Catalogs of convergences, another pervasive evolutionary phenomenon, have been used to downplay that role, the argument being that deterministic selection drives lineages to a favored phenotype regardless of initial states or random interference (e.g. Conway Morris 2003; Vermeij 2006). However, convergences also derive in part from the contingent, limited developmental and thus evolutionary capabilities of lineages confronted by similar environmental challenges (e.g. Wimsatt 2001; McGhee 2011; and for mechanisms extending beyond shared adaptive regimes and developmental limits, see Stayton 2015a, b). In a famous example, *Anolis* lizards converge on limited set of limb configurations and body sizes related to microhabitat preferences on Caribbean islands (Losos 2009), but the equally striking convergence patterns of their skulls do not map onto the postcranial ecomorphs (Sanger et al. 2012), and many mainland species do not readily fall into the island limb/size associations (Schaad and Poe 2010, though see Moreno-Arias and Calderón-Espinosa 2016), suggesting a more complex overall pattern of selection and contingency.

Further, convergences are almost always inexact, so that later modifications and elaborations are unlikely to be equivalent functionally or in subsequent evolutionary potential. The squid camera-eye cannot be mistaken for that of a mammal: the initial formation of light-receptors in protostomes and deuterostomes imposed different structural arrangements, so that we are cursed with a blind spot that cephalopods lack, and we have a light-refracting cornea that cephalopods lack (Serb and Eernisse 2008). Or viewed another way, when selection favored toothlike structures in modern birds, they did not produce true teeth similar to those of their therapod ancestors, but evolved serrations on the keratin sheath covering and extending the beak bones, or, in one extinct Cenozoic clade, novel bony projections on the jaws (Louchart and Viriot 2011). Part of the tooth-development pathway is retained, but key enamel-protein genes were disabled by various mechanisms in the common ancestor of modern birds (Sire et al. 2008; Meredith et al. 2014), so that re-introduction of teeth would constitute convergence, i.e. arrival at a given phenotype from different starting points. [The re-appearance of mandibular teeth in frogs (Wiens 2011) would be a good target for a similar gene-function analysis.]

Convergences are yet more incomplete at the clade level: even the celebrated convergent diversifications of Australian marsupials and placental mammals include on one side kangaroos and koalas lacking close placental analogs, and on the other bats and elephant seals lacking close marsupial analogs. Alternative solutions to a given evolutionary challenge also abound, both within clades (e.g., the greater disparity among the 10 independent origins of durophagy in moray eels than among their various ancestors, Collar et al. 2014), and among clades (e.g. extraction of wood-boring insects by woodpeckers, tool-using finches, and grotesquely elongated digits of the aye–aye lemur; May 1978, p. 172; McGhee 2011, pp. 140–142; and for an extinct, independently evolved instance of digit elongation, see Koenigswald et al. 2005). Finally, as Sterelny (2005) argues, subjective equivalencies can mask profound evolutionary differences, as with human agriculture vs the “agriculture” practiced by leafcutter and other ants (classed as convergence by Conway Morris 2003, p. 198).

A more effective meta-analytical approach to evolutionary contingency cross-tabulates apparent convergences against phylogenetic distance. If contingency is important, increasing phylogenetic distance should erode inherited similarities in phenotype, evolutionary-developmental capabilities and limitations, and biotic and abiotic pressures, and the frequency of convergence should decline accordingly (Ord and Summers 2015). In a pioneering analysis, morphological convergences are heavily skewed toward closely related taxa, in a way that functionally equivalent but divergent phenotypes, such as the woodpecker equivalents above, are not (Ord and Summers 2015 see also Losos 2010). These results show, if nothing else, another way in which phylogeny informs models for any of the macroevolutionary currencies.

Convergences may also provide a framework for another approach to contingency. The temporal gap between the advent of a favorable environment and the appearance of a novel adaptation to that environment might be a measure of the role of contingency in a given evolutionary transition (Foote 1998). Rigorously pinpointing the onset of the environmental factor that sets the start of the waiting time is difficult, but for potential examples in microevolutionary experiments and macroevolutionary biotas, see Blount et al. (2008, 2012) and Dick et al. (2009), respectively. Such new directions require more intensive attention to quantifying the strength and frequency of convergence (see Speed and Arbuckle 2017).

Regarding environmental context, the history of biotic and abiotic environments is one of incessant, episodic change, both cyclic and unidirectional, and these dynamics also impose contingencies. Consider, for example, the scope for evolution accessible to a set of novelties in a stem bilaterian, if acquired before or after oxygen reached levels that could sustain the formation of multicellular bodyplans, collagen, and active aerobic metabolism (see Erwin and Valentine 2013). Although such considerations might seem to explode macroevolution into a welter of unique events, the search for generalizations, models, and ultimately theory is paramount, and has repeatedly yielded ideas of considerable explanatory power. One effective strategy is to accept the rare event as part of the fabric of macroevolution, and aim to understand the differential response of clades having different properties to those events. This approach can be pushed another step further: if clades differ in sensitivity to external events (yet another conceptualization of contingency according to some, see Inkpen and Turner 2012), then they may differ in the role played by contingency in their dynamics: perhaps evolution is more contingent in the volatile ammonoids and more deterministic in the phlegmatic bivalves, at least outside the huge perturbations of the “Big Five” mass extinctions.

In a very different approach, diversification models fitted to the fossil record of one or more clades can attempt to strip out the influence of specific events. For example, Sepkoski (1996) modeled long-term diversity trends to argue that the end-Permian extinction merely hastened, but was not the exclusive driver of, the replacement of brachiopods by bivalves as dominant shelly invertebrates of the world’s seafloors. As with the phylogenetic-distance approach above, one can argue about the details of this analysis, but the approach is an intriguing one that deserves to be revisited.

## The Origin of Variation

Any approach to macroevolution must account for the distinctly *nonrandom* production of phenotypic variation at large spatial and temporal scales. This nonrandom variation (sometimes termed anisotropic) occurs at multiple hierarchical levels, in all currencies, and is manifest throughout the history of life from the initiation of the evolutionary process in protocells, through the burst and diminution of novelty production associated with the Cambrian explosion, to environmental regularities in the origin and later expansion of lesser, post-Cambrian innovations. These patterns, and their underlying mechanisms, require attention because the starting point for most theories of variation—sometimes acknowledged as an operational simplification—has generally been *random* mutational inputs at all levels. The next assumption is generally that all traits are underlain by many genes of small additive effect that mutate independently, with the probability of an increase in fitness inversely related to the magnitude of their phenotypic effects (Fisher 1958; see Tenaillon 2014 for a recent treatment). Elaborating further, genes interact to affect multiple traits, and the extent and apparent randomness of these pleiotropic effects, relative to the attributes of the phenotype, increases the probability that a mutation is deleterious. Such a model is powerful for short-term population studies. However, when considering large-scale evolutionary change, these assumptions must be relaxed or modified—not towards older models of macromutation and evolutionary saltation, although events such as genome duplication and acquisition of endosymbionts might represent modern incarnations of such discontinuities (Futuyma 2015), but to incorporate growing knowledge of the relation between development and evolution. The complexity of the genotype-to-phenotype map undermines any direct correspondence between the differential probabilities of change at the genetic level and the nonrandom probabilities of change at the phenotypic level (see Lynch 2007a, b; Wagner 2011 on “evolvability” of genotype networks; and Plucain et al. 2016 for an experimental cross-level study). On top of this potential for nonrandom mutation, another level of nonrandom variation is interposed between the genome and the organism by development, with its complex interactions involving multiple genetic pathways, cells, tissues, and attendant epigenetic effects (Raff 1996; Arthur 2004, 2011; Wagner and Zhang 2011; Gerber 2014; Nocedal and Johnson 2015; and many more).

### Control Hierarchy

A starting point for a macroevolutionary view of variation is the now-commonplace observation that development is governed by semi-hierarchical networks of genes. These gene regulatory networks (GRNs) are *semi*-hierarchical because, although much information on the time, place, and intensity of gene expression flows from high-level control genes through multiple intermediate steps to batteries of genes at the peripheries of these networks, GRNs contain feedbacks from lower levels within the network that can modulate expression of higher-level genes (e.g. Carroll et al. 2005; Peter and Davidson 2015; Rebeiz et al. 2015; Valentine and May 1996 view these pathways as trees rather than hierarchies in the strict sense, because downstream genes are not physical constituents of upstream ones, see also Tëmkin and Eldredge 2015). This structure is widely held to affect the probability of different transitions, although the specifics are not sufficiently understood to ground a robust theory: some alterations are more readily achieved than others, in part owing to the higher or lower position of different regulatory nodes within a network, but also via connections to preexisting and novel circuits near the network periphery (Davidson and Erwin 2009; Payne and Wagner 2013:; Rebeiz et al. 2015). Thus the probability distribution of the raw material for evolution in a genotype or phenotype space—the inputs presented to selection and other sorting processes—is not isotropic, but uneven, skewed, or channeled. This probabilistic approach to developmental constraint, with some evolutionary directions absolutely unavailable and others accessible to varying degrees (Maynard Smith et al. 1985; Schwenk and Wagner 2004; Klingenberg 2005; Hallgrímsson et al. 2009, 2012; Gerber 2014), may enable stronger mechanistic connections between development and differences in clade behavior in morphospace (Salazar-Ciudad and Jernvall 2010; Gerber 2014 and references therein). That said, we know little about which developmental differences tend to give rise to macroevolutionary ones. For example, transcription-factor binding sites evolve significantly more rapidly in mammals than in *Drosophila*, perhaps because of strong differences in effective population sizes (Villar et al. 2014), but whether these contrasts translate into systematic differences in phenotypic evolution has not been addressed.

### Modularity

Also widely recognized is that development, and therefore the evolution of development, is modular, i.e. organized into semi-independent regions, such that changes in gene expression in one module are more likely to affect GRNs, and ultimately the phenotype, of that module than of other modules. Thus “universal pleiotropy and epistasis” (the rule that every gene affects many traits and traits are determined by many genes of equal and small effect; Fisher 1958; Wright 1968, Ch. 5) is not as pervasive or chaotic as often assumed (see Wagner and Zhang 2011). Indeed, pleiotropy within phenotypic modules can evolve to promote adaptive covaration of related traits, as appears to be the case in flowers of several model systems (Smith 2016). Nonetheless, the discreteness and long-term stability of developmental modules, the consistency of their association with functional modules, and the relation between modules of molecular circuits and sets of phenotypic characters that covary in modular fashion, are still subject to much debate and research, including, of course, how functional, developmental, genetic, and evolutionary modules map onto one another, if and when they do (e.g. Hallgrímsson et al. 2009; Ross 2013; Goswami et al. 2014; Klingenberg 2014; Wagner 2014; Melo et al. 2016). The field has yet to explore or even enumerate the macroevolutionary implications of the alternatives encompassed by the range of current approaches to modularity. For example, modules have been treated on one hand as semi-autonomous developmental and phenotypic compartments (e.g. Klingenberg 2014; Wagner 2014), and on the other as overlapping sets of signaling pathways that may overwrite or obscure each other’s effects to more indirectly impose a phenotypic covariation structure within bodies (e.g. Hallgrímsson et al. 2009). The two perspectives may in part involve differences in analytical resolution and relative emphases on the details of developmental processes, but the key macroevolutionary question is whether these different views on the organization of developmental systems yield different predictions for the phenotypic dynamics of clades over long timescales.

The fact that mosaic evolution is entrenched in our textbooks reflects the ubiquity of some form of phenotypic modularity (to use the agnostic term as in Magwene 2001; Ross 2013), as does the bland statement that every taxon is an amalgam of derived and primitive characters: there is, after all, a developmental story behind the incorporation of every apomorphy into a functional phenotype. Modularity and its converse, integration, may well influence phenotypic transition probabilities at any point in time, and, as they clearly do evolve over time, the maintenance, fusion, and parcellation of modules are likely targets of organismic selection that are, in turn, likely to have macroevolutionary consequences by disallowing or promoting particular directions of further change. The evolutionary stability of phenotypic modules has been addressed in a variety of paleontological studies, from trilobites to mammals (Urdy et al. 2013), but analyses of underlying mechanisms have lagged behind, as have more broad-based evaluations of their macroevolutionary roles (but see Haber 2016 and Hu 2016). Increased modularity may often promote phenotypic diversification (e.g. Vermeij 1974; Raff 1996; Klingenberg 2005; and many others; and see Mondragón-Palomino and Theissen 2008 on parcellation [i.e. splitting of modules] as key to orchid diversification), but this effect is clearly not universal, and quantitative-genetic models indicate that modularity may not be the only genomic architecture that promotes evolvability, depending on factors such as mutation rates (see Pavlicev and Hansen 2011 on potential tradeoffs). Again, empirical work is needed to determine how those factors interact on macroevolutionary scales.

### Tinkering

With the stunning discoveries of conserved developmental pathways across all eukaryotes and especially across Metazoa, many authors have emphasized that much phenotypic change may derive from “tinkering” with development, i.e. small modifications in the timing, location, or combinations of developmental events (Carroll et al. 2005; Lieberman and Hall 2007; Shubin et al. 2009; Wagner 2014). For example, rapid and repeated reduction of the pelvic skeleton in three-spine sticklebacks evidently stem from changes in a regulatory element of the *Pitx1* locus (Shapiro et al. 2004). Such tinkering is of course facilitated by developmental modularity, and can generate a range of phenotypic outputs, from the imperceptible to the dramatic. Thus, new structures need not evolve from scratch, but can arise by modifying or re-deploying ancient GRNs; even highly polygenic structures can shift phenotypically—in certain directions—by altering pre-existing GRNs, and not only by mutations in protein-coding genes. In a further step away from standard microevolutionary models, the hierarchical perspective also makes clear that discontinuous phenotypic variation can be underlain by continuous variation in underlying developmental processes (e.g. Polly 2008; Hallgrímsson et al. 2012).

### Epigenetics

GRNs, and other levels of control such as chromatin and noncoding RNAs, can be responsive, within limits, to extrinsic signals that ranging from interactions among modules in developing embryos to environmental factors such as nutrient levels or temperature. These epigenetic responses, using the term in its original, broad sense, promote the incorporation of altered modules into an integrated, functional phenotype (Hallgrímsson and Hall 2011), and create the potential for adaptive phenotypic plasticity. Examples are increasingly abundant, perhaps the most famous still being Slijper’s goat, born bipedal and whose unusual posture was evidently accommodated by other aspects of the musculoskeletal and nervous system (Rachootin and Thomson 1981; West-Eberhard 2003; for references to controlled experiments on other mammals, see Shi et al. 2007). Such instances are not claims about specific evolutionary transitions but illustrate the latent potential to accommodate heritable changes in one system through epigenetic responses of other systems. Findings at the molecular level corroborate this point. For example, the three-dimensional form of Galapagos finch beaks is orchestrated by two GRNs that mobilize the hundreds of genes that produce the structure, and experimental up-regulation of one of the key genes in the chick produced the expected enlarged beak (demonstrating the genetic architecture underlying this complex structure), which was successfully integrated into the head (demonstrating how epigenetic interactions accommodate modular change) (Mallarino et al. 2011; see also Kirschner and Gehrhart 2010; Lamichhaney et al. 2015).

Genetic assimilation, the genetic fixation of an environmentally induced phenotype by selection for stable expression of the trait, has long been suggested to have macroevolutionary potential. (The broader term *genetic accommodation* refers to any genetic change regulating the expression of initially environmentally dependent traits; it thus includes not only decreases in phenotypic plasticity as in genetic assimilation, but increases in plasticity, and a shift in reaction norm with an unaltered amount of plasticity [West-Eberhard 2003, 2005; Ehrenreich and Pfennig 2016].) Extending beyond Slijper’s goat and other intriguing anecdotes, experimental and field evidence attest to the capacity of genotypes to produce and stabilize adaptive phenotypic variety elicited by internal and external cues, in ways that match fixed variation in related populations (for reviews see Pfennig et al. 2010; Moczek et al. 2011; Schlichting and Wund 2014; Ehrenreich and Pfennig 2016). Genetic assimilation has almost certainly occurred in a number of cases that would generally be classed as microevolutionary. The question is whether and when such changes underlie more dramatic shifts in form and function, and to push potential examples beyond consistency arguments. For example, when *Polypterus* (a basal ray-finned fish with functional lungs) is reared experimentally on land, the neck and shoulder skeleton is modified in ways resembling those of early tetrapods (Standen et al. 2014). This finding lays the foundation for the next level of corroboration for the hypothesis that the resemblances represent the actual course of evolution to the tetrapod condition, e.g. testing for comparable patterns of plasticity in more direct tetrapod ancestors and the direction of changes in plasticity along that lineage, and for the genetic basis of the differences in pectoral girdle development among the clades; and more generally working towards a richer understanding of the genetic and developmental mechanisms that stabilize plastic responses via evolution in the regulation of a trait’s expression. Thus, as in many other issues in macroevolutionary developmental biology, assessing the role of genetic assimilation will require comparative dissection of developmental processes of multiple species, each set of species placed within a carefully framed phylogenetic context (for further discussion on approaches to testing genetic assimilation, see Moczek et al. 2011; Schwander and Leimar 2011; Schlichting and Wund 2014; Futuyma 2015; Ehrenreich and Pfennig 2016; Levis and Pfennig 2016).

### Populations

Evolutionary theory is beginning to incorporate these insights to explore how the relation between development and evolution can transform classic population-genetic models so that they can be used in a macroevolutionary context. This transformation begins with a more realistic genetic architecture, with a range of effect and interaction sizes and which can itself evolve and be related to the generation of phenotypic variation, is an important component (Hansen 2006, 2012; Rice 2012; Rajon and Plotkin 2013; Badyaev and Walsh 2014). The next step, even more challenging, is to approach the evolution of the genotype-to-phenotype map and its modularity in population- and quantitative-genetic terms (Wagner 2010; Pavlicev and Wagner 2012; and see Lande 2009 for a pioneering quantitative genetic approach to genetic assimilation). We are still far from integrating such models with, for example, the behavior of clades in morphospace, but the outlines of this integration are beginning to take shape. One neglected strategy would be to adopt new model developmental systems that can combine well-characterized population genetics with phenotypes traceable from the present day into a high-quality fossil record. This will not be easy: the classical developmental models were selected with a flagrant disregard for paleontological relevance, although mouse and sea urchin may afford a foundation for potential terrestrial and marine systems. (Urchin workers will likely need to shift their sights to heart urchins and sand dollars, which form the bulk of the echinoid fossil record.) Increasingly sophisticated experimental work on gastropods and bivalves (e.g. Henry 2014; Wanninger and Wollesen 2015; De Oliveira et al. 2016) offers enormous potential for linking phenotypes, genetics, and development via an exceptionally rich record that includes fossilized shell ontogenies.

The venerable framework of the fitness landscape may prove to be useful in further linking population genetics to the origin of variation within a macroevolutionary framework. However, a model landscape whose topography is determined by the fitness of genotypes becomes increasingly more rugged as it becomes more realistic for large spatial and temporal scales. History—and anisotropic variation around starting points—has little effect on the behavior of populations on smooth landscape with a single fitness peak: they will reliably converge on that peak, albeit at different rates (Lachapelle et al. 2015). In contrast, a rugged landscape, with multiple peaks and valleys as Wright (1931) envisioned, will hinder access to all but one or few local peaks owing to maladaptive gene combinations between the peaks, so that the contingencies of starting position and the dynamics of the landscape itself (as the fitness of gene combinations shifts through time) become important.

Such rugged fitness landscapes are almost certainly the rule, and are often conceptualized in phenotypic terms as “adaptive landscapes” (Simpson 1944; Arnold et al. 2001; Bell 2012; Hansen 2012). When phenotypes are under selection to satisfy multiple requirements (e.g. feeding, growth, reproduction), tradeoffs reduce absolute fitness but allow different trait combinations to be roughly equivalent (Niklas 1994, 2009; Marshall 2014). Here too, chance and history become increasingly important with increasing ruggedness, as lineages tend to be confined to the peak nearest their starting location. Many have suggested that the developmental factors discussed here offer potential mechanisms for crossing what would otherwise be fitness valleys by phenotypic changes coordinated among parts; proximity of two points in phenotypic space need not map closely to evolutionary-developmental accessibility (Gerber 2014). Further, in the most extreme view, developmental coordination among traits can make fitness valleys disappear. The corollary to this potential, however, is that the patterns of clade deployment within a morphospace (e.g. the uneven distribution of species or higher taxa through the space) cannot be taken as a map of the adaptive landscape, because that density is a function not only of fitness but accessibility given the starting points of clades, the properties of their developmental systems, and as discussed below, factors controlling speciation and extinction rates that need not be closely tied to fitness differences at the organismic level. The adaptive landscape metaphor may seem too abstract and simplified to be of much use, even heuristically (e.g. Pigliucci and Kaplan 2006), but the tradeoff approach to landscape ruggedness has been profitably applied to fossil ammonoid cephalopods (Raup 1967, using different terms), early land-plant evolution (Niklas 2004, 2009) and the Cambrian explosion (Marshall 2006, 2014), and has potential for further refinement and application.

### Species Within Clades

The origin of variation within populations has received more attention than the generation of phenotypic variation among species, but such species-level inputs into the evolutionary process, and how they are translated into net change at the clade level, can reflect the direct and indirect linkages between micro- and macroevolution. Two basic issues must be addressed: first, how the developmental system of organisms governs (or not) the direction of species movement through phenotypic space, and second, the tempo and mode of such changes.

#### Direction

For species within clades, one problem is how aspects of a phenotype or its underlying GRNs and its developmental modules are related to the probability density function of phenotypic change around a given starting point. A simple approach would be to take the cloud of phenotypic variances and covariances existing around that starting point as the probability density function. This strategy, rooted in quantitative genetics (e.g. Lande 1979; Arnold et al. 2001; Melo et al. 2016), has been applied successfully to, for example, populations within a species of stickleback fishes (Schluter 1996), the generation of species within a genus of ostracode crustaceans (Hunt 2007b), and patterns in the reduction of digit number across the tetrapod clade (Shubin and Wake 1996). Not all studies of such within-versus-among species variation can distinguish the raw inputs of developmental systems from the selective processes that filter that variation (but see Alberch and Gale 1985 for the classical experimental approach to tetrapod digit loss, and Pieretti et al. 2015 for an overview of the molecular underpinnings of clade-specific patterns of digit loss). Further, evolution along “lines of least resistance” as inferred from patterns of phenotypic variation, need not indicate the operation of strong intrinsic constraints in all cases (Conner 2012; Melo et al. 2016). Nevertheless, these observations suggest a potential first-order hypothesis for macroevolutionary analysis applicable to both extant and fossil systems at this level: new species within a clade are most likely to originate in the existing directions of variation within each parent species.

This proposition can be related to an influential explanation for the association of phenotypic change with speciation, the hypothesis that speciation stabilizes otherwise-transient phenotypic variation by severing or attenuating gene flow to a local population (Futuyma 1987, 2015). Such a process may also account for correlations between speciation and both clade-level phenotypic evolution (e.g. Rabosky et al. 2013) and rates of molecular divergence (Pagel et al. 2006; Venditti and Pagel 2010; Lancaster 2010; Lanfear et al. 2010; Dowle et al. 2013; Ezard et al. 2013; Bromham et al. 2015), although the causal direction of these correlations remains controversial.

A model relating the direction of speciation in a phenotype space to within-population variation has a rich set of implications for clade-level dynamics. The anisotropic nature of potential variation around any species in morphospace means that, as noted above, proximity of other points in that space is a poor indicator of immediate accessibility (Gerber 2014). Integrating this appreciation of evolutionary accessibility to analyses of clades in morphospace will enhance the integration of development with macroevolution. Thus, understanding how species-level transitions relate to the stability of specific structures or regions of the organism, e.g. a matrix of phenotypic covariances, will be another significant step (for one approach, see Polly 2004). The more similar the probability density functions around all the species in a clade—a little-evaluated possibility—the more likely the clade as a whole will generate a directional trend via successive speciation events.

At the clade level, evolutionary changes must often impose further directional restrictions and biases, however. Thus, aquatic amniotes retain many ancestral traits betraying their terrestrial ancestry. Birds remain committed to an archosaurian, oviparous reproductive mode (in contrast to lizards, snakes, and even fishes and frogs that have evolved viviparity), perhaps not surprising in flying birds that must fare better when the embryo is left on the ground in a protective eggshell, but equally pervasive in flightless birds. Thus, the probabilistic view of evolutionary constraint adopted here cannot be static, but must incorporate how prior steps channel future ones, and thus cuts to the essential role of history in shaping clade trajectories (for a worked example see Aigler et al. 2014 on tooth loss in cypriniform fishes). Unfortunately, a clade’s deployment in morphospace in the absence of developmental data can falsify a constraint hypothesis but is insufficient to prove one. Raup (1966) emphasized that some portions of morphospace are physically untenable, but those vacancies are theoretically less interesting than the feasible but unoccupied portions of the space. The fossil record is replete with phenotypes absent from the modern biota, from giant ground sloths and carnivorous kangaroos to uncoiled nautiloids, coiled oysters, and echinoids with proboscis-like test extensions, each demonstrating the evolutionary accessibility of currently vacant portions of morphospace. One could argue that extrinsic forces are responsible for each of the aforementioned gaps in modern clades, but those missing phenotypes may still reflect intrinsic constraint (with past forms no longer accessible from younger starting points owing to developmental changes), or pre-emption owing to biotic factors such as competitors, or even the waiting time expected between recursions to relatively improbable trait combinations. A similar approach to missing phenotypes, phylogenetic rather than temporal, considers features or functions accessible to some clades but apparently not to others (Vermeij 2015): for example, photosymbiosis and chemosymbiosis are widespread among invertebrate phyla but absent in echinoderms. Such an approach can sharpen hypotheses on impediments such as developmental factors or energetic tradeoffs that might underlie the uneven distribution of certain adaptations.

A considerable body of theory exists on the potential of clades to differ in evolvability, i.e. their ability to respond to directional selection (e.g. Hansen 2006; Kirschner and Gerhart 2010; Wagner and Draghi 2010; Wagner 2010, 2014; Sterelny 2011; Minelli 2016), but rigorously guiding that theory with data has been difficult. Evolvability can be conceptualized in terms of a clade’s ability to generate heritable variation, but a neat separation of evolvability and environmental influences is difficult when plasticity comes into the picture (Brigandt 2015). With respect to macroevolution, evolvability must be concerned not just with the instantaneous response to selection but with sustained responses, and thus the lineage’s ability to accommodate and field variation over many rounds of selection and change. At this scale, evolvability might be quantified operationally by the net amount of morphospace traversed or encompassed by a clade per species over time relative to another clade. Ultimately, however, it will need to reflect back to the nature of the genotype-phenotype map as mediated by development; and, because the macroevolutionary currencies are not precisely correlated, an increase or shift in morphospace occupation need not be accompanied by similar gains or changes in functional diversity. A common-garden design—i.e. analysis over a specified time interval within a single biogeographic province—is needed to hold some external variables constant, but may be insufficient. For example, co-occurring sister clades of neotropical fishes have each traversed roughly the same amount of morphospace since separation, with one ricocheting within narrow bounds—so that total change greatly exceeds net change—and the other diffusing freely from its starting point (Sidlauskas 2008), but the role of development and ecology in this apparent contrast in evolvability cannot be separated without additional data (see Hopkins and Smith 2015 on the contrasting dynamics in morphospace of regular and irregular echinoids).

#### Tempo and Mode

**Fig. 1:**
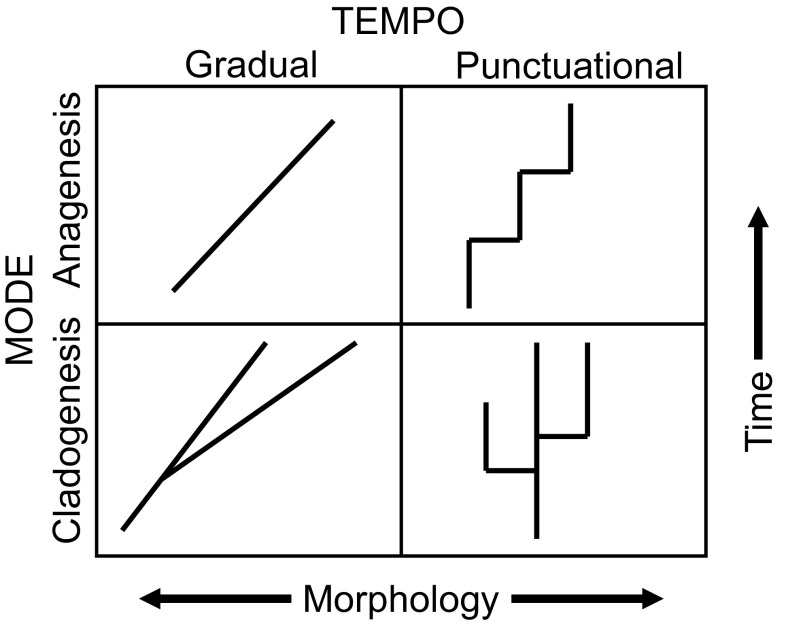
Evolutionary tempo and mode at the species level. The *upper left quadrant* is classic phyletic gradualism, the *lower right* is punctuated equilibrium. All combinations are known in the fossil record. From Jablonski (2007)

Regardless of the biases in the direction of evolutionary change within species, the dynamics of phenotypic change among species has drawn much attention. When the axes are defined in terms of tempo (continuously gradual vs static with punctuations) and mode (cladogenetic, i.e. branching, vs. anagenetic, i.e. non-branching), the famous punctuated equilibrium/phyletic gradualism end-members (Eldredge and Gould 1972) simply become two cells in a matrix of models for evolutionary change: punctuated cladogenesis and gradual anagenesis (see Fig. 1). (“Punctuated equilibrium” has become a blanket term for any pattern of sudden change at any hierarchical level, and in any system from biology to linguistics to education policy, but should be restored to its original intent, a pattern of phenotypic change at the species level over geologic time.) Synthetic analyses have shown that all of the end-member patterns occur in the fossil record, plus intermediates and switching among tempos and mode over time, with sustained directional change being the least frequent (Hunt 2007a; Hunt et al. 2015). Although patterns consistent with random walks are recorded, the temporal scaling and rates of change are generally intermediate between random walks and stasis, and closer to the latter (Hunt 2013). Evolutionary stasis has been defined in many ways, but the key feature is statistically negligible *net* phenotypic change at the species level, i.e., a lack of directionality rather than of evolutionary lability, and species in the fossil record often show high total rates of evolution while accumulating little net change (e.g. Stanley and Yang 1987; Gould and Eldredge 1993; Jablonski 2000; Gould 2002). Detection of cladogenesis requires temporal overlap between sister taxa, or ancestor and descendant, and improved methods have required re-evaluation of some putative instances of anagenetic transitions (e.g. Hull and Norris 2009). However, as long noted (Gould and Eldredge 1993; Jablonski 2000), punctuated models of phenotypic evolution do not require that all speciation events incite phenotypic change, but that net phenotypic change is mainly associated with speciation. The challenge now is to develop and test theory that accounts for the distribution of species-level tempo and mode across the tree of life (Jablonski 2000, 2007).

Various authors have proposed that different genetic population structures or environmental tolerances pre-dispose clades to different evolutionary tempos and modes, and that different habitat types—for example surface ocean vs shallow seafloors—are more likely to promote different patterns among otherwise-disparate clades (Jablonski 2008b). The many hypotheses, not all mutually exclusive, for the mechanism behind species-level evolutionary stasis (e.g., Bell 2012; Arnold 2014; Hunt and Rabosky 2014; Futuyma 2015), require tests that separate the alternatives to assess their relative frequencies. This enterprise is aided by the application of models that can be assessed by Bayesian or information-criterion approaches, most commonly Brownian motion (BM) and Ornstein–Uhlenbeck (OU) models (Lande 1976; Hansen 1997), or their combination (Hunt et al. 2015). These models are valuable, but are not diagnostic of specific evolutionary processes. For example, a species can fit a BM model under drift, tracking of randomly varying environments, or the interaction of many independent forces (e.g. Pennell et al. 2015). Fit to an OU model, where the probability of change decreases with distance from a specified trait value, is often conceptualized as fluctuation around an optimum, but other intrinsic and extrinsic factors invoked to explain stasis (see Futuyma 2015) cannot be ruled out. And of course shifts from one stable position to another can be mediated by many mechanisms, from drift (an early favorite), to environmental tracking by an isolated subpopulation, to correlated responses to selection on one character that drags others with it (e.g. Hansen 2012).

### Evolutionary Novelties

The term novelty has a tormented terminological and conceptual history, but some useful distinctions are outlined by Wagner (2014, 2015), drawing on many prior sources. The first distinction is between functional and phenotypic novelty; some have suggested the restriction of “innovation” to the former, and “novelty” to the latter (Müller and Wagner 1991; Love 2006; Wagner 2014, 2015; Wainwright and Price 2016; for different uses of these and related terms, see Erwin 2015b). The origin of new functional capacities, such as flight or endothermy, is often related to morphologic change of course, but the magnitude of functional divergence from an ancestor may be only loosely related to the magnitude of phenotypic divergence (e.g. Wainwright 2007; but see Jablonski 2008a). Wagner (2015) notes that analysis of functional divergences involves a significant emphasis on the viability of transitional forms between two discrete functions (running and flying, for example). Such an emphasis will be less central to analyzing the mechanisms behind morphological novelties, but remains an important macroevolutionary issue, in terms of the ecology and population genetics of novelty.

A second distinction lies between two types of evolutionary novelty (e.Müller 2003, 2010; Wagner 2014, 2015; see also Moczek 2008 for a thoughtful discussion). Type I entails the origin of a genuinely new feature or body part lacking a structural homolog in the ancestral clade, such as the vertebrate head—a developmentally individuated structure (Wagner 2014; adopted and discussed by; Erwin 2015b). Type II entails the radical transformation of an existing body part, such as forelimbs to wings or flippers. “Radical” here indicates an evolutionary and developmental commitment to these modifications such that a reversion to the ancestral form is highly unlikely. Thus, while the fin to limb transition was surely one of the most significant morphological shifts in Earth history, it involved modifications to pre-existing appendages. Nonetheless, when vertebrates return to a purely aquatic existence, they never revert to true fins, with fin rays, but modify the tetrapod limb plan in various ways. As in most biological definitions, the boundaries here are not sharp; beetle horns are novel morphologic structures that involve the *distalless* GRN usually involved in limb development, a “deep homology” (Shubin et al. 2009) that constitutes phenotypic novelty but commandeers an existing developmental pathway. Still unclear are the macroevolutionary consequences of these different types of novelty, e.g. for future range or directions of evolutionary change. Type I novelties might open more evolutionary trajectories to a clade than do Type II novelties (as the vertebrate head surely did, relative to, say, wings), but that expectation has not been tested across a wide range of features, and beetle horns (=Type I) fall short of, say, bird wings (=Type II), in generating diversity in any of the macroevolutionary currencies. A set of formal sister-group comparisons is needed.

Many authors have suggested that true novelties may have a different developmental basis from less dramatic modifications of existing body parts and physiological processes (recently Davidson and Erwin 2009; Müller 2010; Wagner 2014). Such hypotheses—still speculative—need not involve Goldschmidt-style macromutations (i.e. extensive genomic upheavals), but posit that novel cell types or quasi-independent developmental modules are most likely to derive from a particular set of mutations: origin of new gene-regulatory sequences (e.g. from transposable elements), new transcription-factor functions, new microRNAs changing spatial patterns of gene expression—all of which could have trivial or strong phenotypic effects, depending on where and how they occur. If this hypothesis is even partly correct, the next question is whether such a link between phenotypic novelty and genetic mechanism imparts macroevolutionary pattern: do these mechanisms operate homogeneously in time, space, and among clades? For example, if mutations driven by transposons are most prevalent with the invasion of a new retrovirus (Wagner 2014, pp. 207–208), or during interspecific hybridization (see Abbott et al. 2013), is the frequency of novelty indirectly related to genetic population structures, or the number of congeneric species encountered by a species over time?

Two additional aspects of phenotypic evolution have sometimes been placed under the rubric of evolutionary novelty. One is the expansion of clades within morphospace. However, the occupation (or re-occupation) of portions of such a space can involve combinations of primitive and derived character states, or translation of clades along quantitative adjustments in shape, or even allometric trajectories where shape changes are simple functions of body size. These shifts may or may not involve significant developmental alterations. An even more inclusive definition of novelty encompasses all the derived characters (apomorphies) that demarcate units across the taxonomic hierarchy and constitute the character-state matrices of formal cladistic analysis (e.g. Charlesworth 1990). Such breadth renders the term almost meaningless from a macroevolutionary perspective. However, a valuable, neglected contribution to macroevolutionary theory made by character-state matrices is their demonstration that homoplasy (convergence and parallelism) is truly pervasive across the tree of life. Every measure of character-state conflict (Consistency Index, Retention Index, et al.) quantifies this homoplasy, and the raw data matrices map phenotypic hot spots and cold spots in these effects. Of course, the significant frequency of character-state conflict in phylogenies can arise from many non-exclusive factors, from repeated selection among diverse phenotypes to meet specific challenges, to exhaustion of readily accessible evolutionary options (Wagner 2000; Wagner and Estabrook 2014; Cuthill 2015).

By virtually any definition of the term, evolutionary novelty exploits the modularity and hierarchical control of GRNs. Clearly, changes in the location and timing of developmental events can promote more dramatic and coordinated phenotypic changes than expected from a Fisherian model, and such changes are more likely to be adaptive than the Fisher expectation because they draw on existing ontogenetic pathways (e.g. Gould 1977, 2002). Heterotopy, changes in the location of developmental events, has increasingly been appreciated for its evolutionary potential (Zelditch and Fink 1996; Baum and Donoghue 2002). Heterochrony, changes in the timing of developmental events, has been dismissed in macroevolutionary terms as simply drawing on existing variation (e.g. Zelditch and Fink 1996), but two points ameliorate that view. First, heterochrony in clades having multi-phase life cycles can yield significant ecological changes, i.e. functional innovation. Heterochrony in such lineages can also alter dispersal abilities (as a byproduct or even as the direct target of selection) that in turn influence genetic population structures and geographic range sizes, thereby affecting origination and extinction rates—excellent examples of upward causation in action. If heterochronies are initiated by relatively simple genetic changes, we might expect such transitions to occur repeatedly, as seen in permanently aquatic, paedomorphic salamanders (Johnson and Voss 2013), and permanently planktonic, paedomorphic gastropods (Teichert and Nützel 2015).

The second evolutionary role of heterochrony lies in local heterochrony. Gould (1977) emphasized global heterochrony, or more precisely the dissociation of sexual maturation from somatic development. However, the modular nature of development permits a much greater array of developmental shifts involving specific structures or regions of the body—just as it does changes in the location of developmental events, giving rise to heterotopies. Such local heterochronies have occurred frequently, again with potential impact on ecology and gene flow. The appendage heterochronies that produced the skeletal structures supporting wings in pterosaurs, birds, and bats are good examples, but many others exist, a striking one being the paedomorphic derivation of bird skulls (Bhullar et al. 2012, 2016) and perhaps feet (Botelho et al. 2015), although one or more episodes of global heterochrony that brought overall miniaturization and paedomorphosis, modified by other, local developmental changes in limbs and other systems, also seems plausible. Thus, while Raff (1996) and others are certainly correct that most evolutionary transformations are not underlain by heterochrony, as was sometimes implied during the renewed wave of enthusiasm for the concept (e.g. McKinney and McNamara 1991), heterochrony is one of the mechanisms linking development to macroevolution (Arthur 2011; Hanken 2015; Minelli 2016). Greater clarity can be achieved through a hierarchical view: many molecular heterochronies do not result in phenotypic heterochrony (e.g. origin of elongated body plans by changes in the molecular “segmentation clock”, Keyte and Smith 2014), and many phenotypic heterochronies lack an underlying change in the timing of gene expression (e.g. paedomorphosis in salamanders via the severing of a hormonal pathway, Johnson and Voss 2013).

The larger challenge is to develop a theory linking developmental changes to the differential behavior of clades and traits over time. Contrasts in rates and patterns of phenotypic or taxonomic evolution are often associated with clade-level differences in development, but are the macroevolutionary dynamics driven by the developmental differences, or do the developmental differences arise later, stabilizing favored phenotypes? Intriguing signposts for theory do exist, if we can argue that the exceptional beak diversity achieved by finch clades relative to other songbirds stems from the simplicity of the GRNs governing their beaks’ three-dimensional form (Mallarino et al. 2011; Fritz et al. 2014), or that flightlessness evolves so frequently in rails relative to other bird clades owing to late ossification of their sternum (Feduccia 1999). Integrated experiments and model simulations can build on these observations to produce stronger links between aspects of development and large-scale phenotypic outcomes. For example, the strength of developmental integration, or its converse modularity, and the eccentricity of phenotypic covariation within populations around a multivariate centroid, appears to influence the differential evolution of skull disparity between mammalian clades (Goswami 2006; Porto et al. 2013; Goswami et al. 2014; Haber 2016). The next step is to test for generality of such patterns, for example in other modules within the tetrapod body.

The differential occupation of morphospace among clades can be a vehicle for addressing many macroevolutionary issues, particularly if it can be coupled with the developmental underpinnings of form. Thus, if beetles can build horns by a re-positioned distalless module, why can’t tetrapods co-opt that module to make dorsal wings, as have sprung from the human imagination many times, from winged horses to dragons? The absence of such wings in the real world—and the fact that tetrapods have always evolved powered flight via modified forelimbs instead—has often exemplified developmental constraint (e.g. Erwin 2007; Losos 2011). Thus, we need a theory that both accounts for the origin of beetle horns and disallows certain other appendages that might use the same core developmental pathway. The answer may lie in epigenetics and the limits to how novel structures are integrated into the developing body, in fundamental differences between protostome and deuterostome development, or in the selective value of incipient structures (i.e. an exaptation scenario for proto-wings in a horse; thermoregulation and sexual selection have been viable alternatives in other contexts). This somewhat absurd example of a missing form underscores ingredients for a macroevolutionary approach to variation. Experimental manipulation of developmentally important genes has begun to probe these limits to development, and we are at the threshold of new advances in this area. Such experiments, potentially in tandem with computational models of developing structures, now can be designed to illuminate the major evolutionary issues (e.g. Harjunmaa et al. 2014; Pieretti et al. 2015).

The macroevolutionary lags discussed above indicate that both the causes and consequences of evolutionary novelty, by any definition, are still poorly understood. Novelties are not reliably tied to ecological opportunity or to taxonomic diversification, as has often been assumed. A basic question is whether the duration of lags or the magnitude of post-lag diversifications can be predicted from some aspect of the novelty itself, of the clades that capture novelties, or of the ecological context in which they arise. Further, even bona fide key innovations (i.e. novelties closely associated with increased diversification) can limit potential evolutionary directions. For example, complex pharyngeal jaws repeatedly promote teleost diversifications via feeding strategies, but by limiting potential prey size evidently interfere with the evolution of piscivorous diets (see Wainwright and Price 2016). One way to evaluate the potential constraining effects of evolutionary transitions, including key innovations, is by analysis of evolutionary consequences of secondary losses of the trait in question. For example toe pads opened ecological opportunities on vertical surfaces for several lizard lineages, but evolutionary rates are higher in lineages that had secondarily lost this feature, which evidently limits ecological opportunities in other ways (Higham et al. 2015).

### Origin of Novelties in Time and Space: Empirical Challenge for Theory

The nonrandom spatial and temporal patterns in the origin of evolutionary novelties, in the broad sense of significant departures from ancestral states in morphology and function, show how important ecology, development, and their intersection are to macroevolution (see Jablonski 2010), but generalizing from these patterns, and probing the underlying processes, has been difficult. At least three broad patterns in the origin of evolutionary novelties (or their rough proxies, higher taxa), need to be addressed.

#### Temporal

The most dramatic pattern is temporal. The Cambrian explosion of animal body plans, and, not coincidentally, the first appearance of most phylum-level taxa, is unmatched in the preceding 4 billion years or the ensuing half-billion (see Erwin and Valentine 2013 and Erwin 2015a on the uniqueness of this event). This geologically brief interval when virtually all fossilizable bilaterian body plans appeared for the first time is perhaps of greatest interest in terms of (a) how eukaryotic development as constructed in the Proterozoic—presumably derived though some combination of stepwise selective modification and frozen accident—has both promoted and limited evolutionary change ever since, and (b) whether phenotypic evolution in the late Proterozoic and the Early Cambrian represent a mode of evolution inaccessible to later genomes, populations and clades, or a unique ecological situation. The authors of a major contribution to this subject (Erwin and Valentine 2013) could not agree on the relative roles of ecological opportunities and feedbacks and “immature” or permissive developmental systems in enabling, and then damping, the unique inventiveness of Proterozoic and Cambrian clades. Absent the invention of a time machine allowing trans-temporal transplant experiments (predictions for swapping modern and early Cambrian lineages under the two alternatives are clear), models integrating development, phylogeny, and the fossil record offer the best hope for progress in this area.

**Fig. 2:**
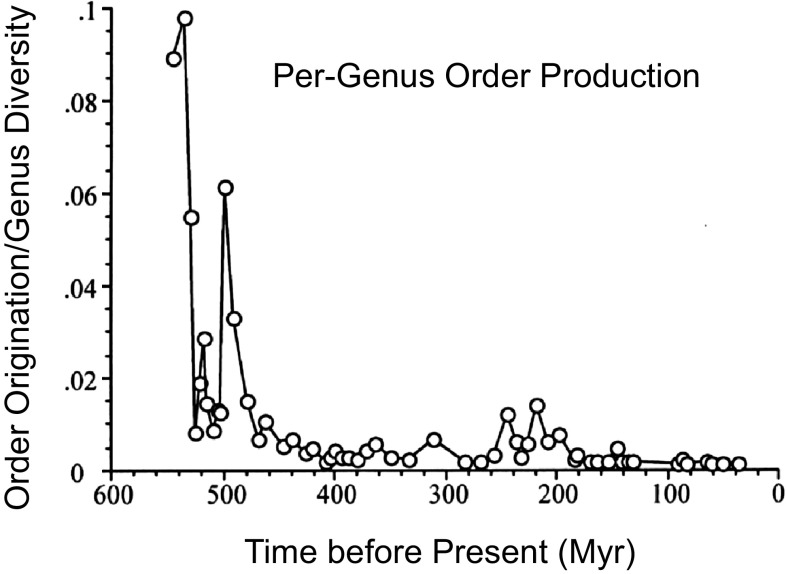
Time- and diversity-dependence in the origin of marine invertebrate orders, normalized by the number of genera recorded globally for each time bin through the Phanerozoic. Courtesy of Gunther Eble, Université de Bourgogne, from Jablonski (2010a)

Less profound evolutionary novelties or innovations have appeared in droves since the Cambrian, from tetrapod digits to the wings, flippers and hands modifying those digits. Mass extinctions, discussed further in Jablonski (2017), clearly promote prolific taxonomic diversifications among the survivors, presumably owing to ecological opportunities, but they bring relatively modest pulses of evolutionary novelty on a per-taxon basis (Fig. 2). Nor are Maynard Smith and Szathmary’s (1995) eight “major transitions” associated with mass extinctions, although seven of them are Precambrian, when extinction rates are poorly known. At least the first six transitions, through multicellularity, evidently pre-date extinctions associated with the extensive “Snowball Earth” glaciations of the Proterozoic (Erwin and Valentine 2013). The timing of the seventh, the transition from solitary individuals to colonies including non-reproductive castes, is uncertain but has occurred repeatedly over the past 700 Myr or more, also with no clear association with mass extinctions. (The origin of human societies may (Szathmary 2015) or may not (McShea and Simpson 2011) represent an eight transition comparable to the others.) The major botanical events—e.g. origin of vascular tissues, secondary growth (allowing the evolution of complexly tiered forests), or flowers—also occur away from recovery phases following the canonical mass extinctions (McElwain and Punyasena 2007; Cleal and Cascales-Miñana 2014), although some might be tied to other physical environmental drivers, such as rising atmospheric oxygen levels. The overall impression is that mass extinctions serve mainly to remove ecological dominants, both in plants and animals, and allow once-marginal clades to diversify taxonomically, functionally, and morphologically. These post-extinction pulses are not trivial: mammals put bats in the sky and proto-whales in the sea within ~10 Myr of the end-Cretaceous extinctions, for example, and such diversifications help buffer clades from future environmental changes, but no fundamentally novel body plans arose in the style of the Cambrian explosion. Thus, regarding evolutionary novelty, macroevolutionary theory must account for this uneven clustering of events, where even the tightest biodiversity bottlenecks, such as the gargantuan end-Permian extinction (which removed ~75% of marine genera and so 80–90% of marine species; Raup 1979; Foster and Twitchett 2014; Stanley 2016) are not followed by Cambrian-like recoveries.

#### Environmental

**Fig. 3:**
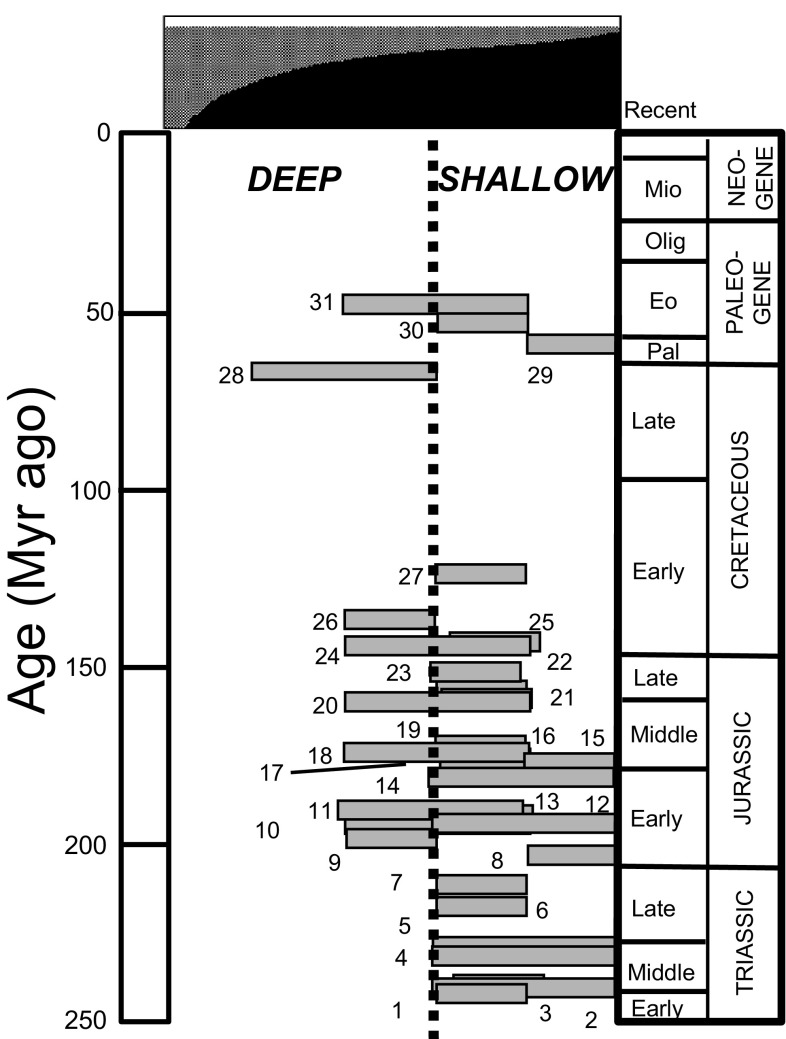
Post-Paleozoic higher taxa (orders) of marine invertebrates having good preservation potential tend to appear first in onshore habitats, generally expanding or shifting offshore over geologic time. Modified after Jablonski (2005b), which also plots the contrasting pattern of orders having poor-preservation potential, which can serve as a sampling control and differs significantly from the onshore–offshore pattern shown here. Orders: *1* Encrinida; *2* Millericrinida; *3* Scleractinia; *4* Isocrinida; *5* Thecidida; *6* Pedinoida; *7* Tetralithistida; *8* Phymosomatoida; *9* Pygasteroida; *10* Cyrtocrinida; *11* Orthopsida; *12* Cephalaspidea; *13* Holectypoida; *14* Cassiduloida sensu lato (basal Neognathostomata *sensu* A.B. Smith); *15* Calycina (Salenioida); *16* Lithonida; *17* Disasteroida; *18* Arbacioida; *19* Lychniscosida; *20* Echinoneina; *21* Sphaerocoelida; *22* Cheilostomata; *23* Milleporina; *24* Spatangoida; *25* Holasteroida; *26* Temnopleuroida; *27* Coenothecalia (Helioporacea); *28* Stylasterina; *29* Clypeasteroida; *30* Echinoida; *31* Oligopygoida. The echinoid orders (numbers 6, 8, 9, 11, 13–15, 17, 18, 20, 24–26, 29–31) could be re-analyzed in the phylomorphospace developed for the group by Hopkins and Smith (2015), but a developmental morphospace is still in very early stages

Environmental patterns in the origin of novelty are also significant. The most extensively documented of these patterns appears to be onshore–offshore differentials in marine invertebrates. Paleontological evidence indicates that higher taxa representing new morphologies associated with substantial functional changes (evolutionary innovations sensu Wagner 2014) preferentially originated in shallow-water environments and later spread across the continental shelf over millions or tens of millions of years (Fig. 3). These dynamics are best-documented for the past 250 Myr (Jablonski 2005b), although some data suggest a similar pattern in some clades and regions in the Paleozoic and even in the midst of the Cambrian explosion (references in Jablonski 2005b; also Harper 2010; Kiessling et al. 2010; Miller 2012; but see Harper et al. 2015 at lower taxonomic ranks), and analogous patterns have been proposed for terrestrial plants (references in Jablonski 2005b). It is unclear what fraction of these evolutionary events represent Type I novelties in the strict, developmental sense discussed above, but what is striking for the post-Paleozoic marine clades is the demonstration that the onshore-origin of order-level taxa—and the invasion of novel portions of morphospace, in the lone phenotypic study (Eble 2000)—contrasts significantly with the first appearances of genera and families, which tend to conform to their clade-specific bathymetric diversity gradients. This discordance across taxonomic levels suggests that the origin of major groups onshore reflects a macroevolutionary tendency that does not scale up simply from within-population genetic or ecological processes (Jablonski 2005b). Further, lags occur in this onshore–offshore dimension: clades take millions or tens of millions of years to spread across the continental shelf, often at low within-habitat diversities, and only diversify later in their histories (Jablonski 2005b and references therein). These phenomena are potentially fertile ground at the intersection of evolutionary developmental biology and macroevolutionary modeling.

#### Biogeographic

Many significant evolutionary transitions have been traced back to the tropics, and paleontological work has confirmed this general tendency for marine invertebrate orders (Jablonski 1993, 2005b; Martin et al. 2007; and see Vermeij 2012 on phenotypic novelty). However, in contrast to bathymetric patterns, both standing diversity gradients and the origins of lower taxa (here, genera) also peak in the tropics (e.g. Jablonski et al. 2006, 2017; Jansson et al. 2013), so that the dynamic is less clear on a per-taxon basis. Paleontological sampling deficiencies in the post-Paleozoic tropics suggest that originations at all levels are higher than directly observed, but a precise per-taxon rate is difficult to obtain (Jablonski 1993; Jablonski et al. 2006); Kiessling et al. (2010) attempt to factor out sampling and conclude that tropical per-taxon rates of genus origination did exceed extratropical rates. Finally, lags appear to operate here as well, in that lower-level clades may remain bottled up in the tropics for millions of years before expanding poleward (Jablonski et al. 2013).

Overall, the uneven temporal and spatial distribution of evolutionary novelty, whether defined broadly in phenotypic terms, or narrowly in developmental terms (Types II and I, above), is difficult to fit into simple probabilistic models predicated on random mutation, species-level phenotypic rates, or trends in species richness. A macroevolutionary amalgam of developmental biology, genetics, and ecology is clearly needed to address this intriguing set of problems. For example, one way to interpret the patterns described in this section are that population-genetic factors create conditions for more profound evolutionary changes in some settings than others. A careful partitioning of novelties as divergent phenotypes vs novelties as traits underlain by newly individuated developmental pathways may be revealing, And of course, if the environment is an active agent in shaping phenotypes and not just a selective filter on genetically fixed phenotypes (as discussed above), then on one hand the configuration and evolution of developmental reaction norms need to be studied more directly in a comparative, phylogenetic framework, and on the other, we might expect certain environments to be especially fertile grounds for evolutionary change owing to mechanisms that have been little-explored in the most branches of ecological developmental biology. In this respect and many others, we still have a long way to go to fully apply Van Valen’s (1973) evocative dictum, “Evolution is the control of development by ecology.”

## Conclusion

Approaches to the origin of phenotypic variation as the raw material for macroevolution have greatly expanded in recent years, thanks largely to advances in developmental biology and in quantitative methods in paleontology and other facets of historical biology, although a more complete integration of these fields is needed. The concepts and data described here are coalescing into a framework for this integration, and great potential is undeniably there. From a macroevolutionary perspective, the most powerful impetus for integration is the discovery of patterns unexpected under the conventional genetic paradigm dominated by phenotypically random mutations of minute effect and universal pleiotropy. The nonrandom distribution of phenotypes in morphospace, through time, and across environments, plays out at multiple hierarchical levels and so is amenable to integrated study in many systems, from the widest, most comprehensive focus to the narrowest and the most particular. The analytical and modeling toolkits in both fields, and at their intersection, are gaining great power. The next step is to hone a macroevolutionary theory that does not simply accommodate the observed patterns but is sufficiently mechanistic and multidimensional to gain predictive power (see also Jablonski 2017).
